# Isolation and Identification of Duck Intestinal Probiotics and Their Effects on the Production and Immune Performance of Pekin Ducks

**DOI:** 10.3390/biom15091217

**Published:** 2025-08-22

**Authors:** Zhigang Hu, Zhuo Zhi, Huiya Zhang, Jie Zhou, Mengmeng Cui, Jianqin Zhang, Dongfeng Xue, Xiaolin Liu

**Affiliations:** 1 College of Animal Science and Technology, Northwest A&F University, Yangling 712100, China; huzg0326@nwsuaf.edu.cn (Z.H.); zz@nwafu.edu.cn (Z.Z.); zhanghuiya@nwafu.cn (H.Z.); zhoujie2000@nwafu.cn (J.Z.); 2285235497@nwafu.edu.cn (M.C.); zhangjianqin0822@nwsuaf.edu.cn (J.Z.); 2 Livestock and Veterinary Workstation in Zhichuan Town, Hancheng 715400, China; poultryduck@163.com

**Keywords:** duck, *Enterococcus faecalis*, immune performance, *Lactiplantibacillus plantarum*, production performance

## Abstract

The purpose of this study was to investigate the effects of duck-derived probiotics added to drinking water on the production and immune performance of Pekin ducks. Two strains with good biological characteristics were isolated from the cecum of Pekin duck and identified as *Lactiplantibacillus plantarum* (*L. plantarum*) and *Enterococcus faecalis* (*E. faecalis*) by species identification. Then, a total of 90 uniformly sized and healthy 7-day-old Pekin ducks were randomly divided into three groups (six replicates per group, five ducks per replicate). Ducks in the control group were fed the basal diet (control group), and those in the experimental groups were fed the basal diet and supplemented with 1 × 10^7^ CFU/mL *L. plantarum* (LP group) and *E. faecalis* (EF group) in drinking water, respectively. The supplementation of *L. plantarum* and *E. faecalis* in drinking water could significantly improve the average daily feed intake (ADFI) and average daily gain (ADG) of Pekin ducks, as well as the live weight, eviscerated weight, half-eviscerated weight, breast muscle weight, and leg muscle weight (*p* < 0.05). Compared with the control group, the duodenal villus height, duodenal V/C (villus height and crypt depth ratio), and ileal villus height were significantly increased in LP and EF groups (*p* < 0.05). Moreover, supplementing the *L. plantarum* and *E. faecalis* significantly improved the immune organ index and serum immunoglobulin A (IgA) content, and reduced the serum immunoglobulin G (IgG) content (*p* < 0.05). They also significantly decreased the number of pathogenic bacteria in the cecum of Pekin ducks and increased the number of *Lactobacillus* sp. (*p* < 0.05). This study indicated that adding duck-derived *L. plantarum* and *E. faecalis* can improve the production and immune performance of Pekin ducks, as well as enhance the structure of their gut microbiota and protect intestinal health. These findings deepen our understanding of the functions of duck-derived probiotics and provide a foundation for their use as feed additives.

## 1. Introduction

The structure and function of the gut microbiota are crucial for the health of poultry, as it has a significant impact on the development of avian intestinal epithelium and the regulation of maintaining intestinal homeostasis (immunity, nutritional digestion, intestinal barrier integrity) [1,2]. Conversely, these functions are essential for optimizing the efficiency of the host in extracting and utilizing energy [3]. Gut microbiota are closely related to poultry productivity, and their interrelationships have been widely studied [4,5]. Microbial dysbiosis can lead to alterations and functional damage at the gene or protein level, ultimately resulting in disruptions in the response of intestinal epithelial cells and the immune system, as well as changes in intestinal metabolites [6,7]. Specifically, when intestinal epithelial cells are damaged, their ability to digest and absorb nutrients declines, ultimately leading to problems such as slow growth, weight loss, and weakened resistance in poultry. Antibiotics mainly exert their effects by inhibiting bacterial cell wall synthesis, enhancing bacterial cell membrane permeability, interfering with bacterial protein synthesis, and inhibiting bacterial nucleic acid replication and transcription. Excessive use of antibiotics can lead to a loss of balance in the gut microbiota, resulting in an increase in the number of yeast (such as *Candida albicans*) and bacteria (such as *Proteobacter* sp., *Staphylococcus* sp., and *Clostridium difficile*). The number of these bacteria in the intestine is usually very small, and their increase can decline in digestive function or the occurrence of gut-related diseases [8,9,10]. As a result, antibiotics can reduce the diversity and abundance of the gut microbiota, causing a decrease in competitive rejection ability. This indirectly disrupts the community structure, thereby interfering with the interactions between microbial species and the complementary system of nutrient metabolism pathways, leading to widespread fluctuations in the gut environment. Even after stopping the medication for a few months, these changes have not been completely reversed [11,12]. Currently, the use of antibiotics to enhance growth and feed efficiency and reduce mortality has been banned in animal nutrition in China.

The word Probiotics is derived from a Greek word and means “life”. It is defined as “living microorganisms that, when given in sufficient amounts, produce health benefits for the host”. Lactic acid bacteria, including several genera such as *Lactobacillus* sp., *Streptococcus* sp., *Lactococcus* sp., *Pediococcus* sp., and *Enterococcus* sp., can tolerate low pH, high salt concentrations, and heat treatment [13]. Lactic acid bacteria can utilize multiple carbon sources for energy metabolism, produce lactic acid through glycolysis, and yield metabolites such as ethanol, acetic acid, and carbon dioxide through the pentose phosphate pathway [14]. Most lactic acid bacteria are probiotic microbes that generate enzymes with antibiotic, anticancer, and immunosuppressive properties [15]. In animal production, lactic acid bacteria are mainly used as feed additives, which have the functions of improving intestinal microbiota balance, promoting growth, and enhancing immunity. Lactic acid bacteria regulate intestinal pH by producing metabolites such as lactic acid to inhibit the proliferation of pathogenic microorganisms and maintain gastrointestinal health [16]. In addition, lactic acid bacteria can also significantly improve the taste of food, strengthen the body’s absorption of nutrients, and reduce blood lipids, cholesterol, and other functions [17]. In order to meet the huge demand for probiotics, it is necessary to conduct new sources of probiotics and extensive routine screening studies, which can screen for new candidate bacteria and conduct efficacy tests in animals for application in the health, feed, meat, or egg production. Ahmed et al. isolated 21 strains of *Lactobacillus* from the chicken gut. These strains have significant acid resistance, bile salt resistance, and antibacterial activity, and they can be used in poultry feed formulations [18]. Suryadi et al. found that dietary supplementation of a novel probiotic comprising microorganisms from cattle rumen and chicken intestine had significant effects on increasing the protein content and decreasing fat content of broiler meat [19]. The first step in selecting potential probiotics is to isolate and identify microorganisms from the gut, feces, and milk of respective animals. It is then demonstrated whether these identified probiotics can improve animal production performance, reduce the risk of intestinal disease, protect animal health, and thus improve product quality and revenue.

The production of meat and eggs in poultry (chicken, waterfowl) plays a significant role in meeting the growing demand for animal protein. China is not only the world’s largest producer of waterfowl (including ducks and geese) but also the largest consumer of waterfowl products, with the total import and export volume of waterfowl products ranking first in the world. In recent years, the duck industry in China has developed rapidly, with obvious increases in the amount of breeding, slaughtering, and meat production. The production of duck farming is gradually developing toward scale, intensification, and industrialization, and the yield and quality of ducks have been greatly improved. Therefore, ensuring the green, healthy, high-quality, and rapid growth of ducks has attracted more and more attention. In 2024, the number of commercial meat ducks sold in China reached 4.22 billion, with a meat production of approximately 10 million tons. Pekin ducks are a famous meat duck breed with high reproductive performance, fast growth rate, and good meat quality. In order to explore the effects of duck-derived probiotics on duck production and immune performance, Pekin ducks were selected as the research objects to screen and identify the intestinal probiotics and detect their biological characteristics in this study. Afterward, it was determined whether probiotics could promote the growth and slaughter performance of Pekin ducks and improve their immune level through feeding experiments, providing scientific references for the application of probiotics in the duck production.

## 2. Materials and Methods

### 2.1. Source of Probiotic Samples

The bacterial isolates used in this study were isolated from the cecum of Pekin ducks. Eight 35-day-old Pekin ducks, raised under the same environmental conditions (net-flat rearing) and free access to feed and water, were selected from Shaanxi Fuqiang Hongtu Animal Husbandry Co., Ltd. (Hancheng, Shanxi, China). The ducks were not fed any antibiotics.

### 2.2. Isolation and Identification of Probiotics from Duck Intestine

After slaughter, 0.1 g of intestinal contents was immediately collected from the cecum and placed in a centrifuge tube containing 10 mL of sterile physiological saline. After sufficient shaking, the mixture was diluted in a gradient to 10^−7^ g/mL. Then, 100 μL of diluted solution was inoculated into an MRS agar plate (DeMan, Rogosa, and Sharpe medium) and incubated for 48 h at 37 °C under anaerobic and aerobic conditions. Colonies with obvious calcium-soluble circles were selected, and then isolation and purification of colonies were carried out by subculturing onto MRS agar 3 times. The morphology of isolated colonies was observed, and a Gram stain test was performed.

The selected colonies were inoculated into bacterial micro-biochemical identification tubes according to the instructions (Hope Bio, Qingdao, China), and the identification results were compared with those in the instructions. Biochemical tests included the catalase test, V-P test, MR-VP test, gelatin liquefaction test, hydrogen sulfide test, and complete biochemical identification test of lactic acid bacteria. DNA extraction of the selected bacteria was conducted by using a DNA extraction kit (TIANGEN, Beijing, China), and PCR amplification of 16S rDNA was performed using bacterial universal primers (27 F: 5′-AGAGTTTGATCCTGGCTCAG-3′, 1492R: 5′-ACGGTTACCTTGTTACGACTT-3′, fragment length 1500 bp). The PCR product was checked by 1.5% agarose gel electrophoresis, purified, and sequenced. The nucleotide sequence was used for sequence identity analysis through NCBI (https://www.ncbi.nlm.nih.gov/; accessed on 18 March 2023) to determine the types of probiotics.

### 2.3. Biological Characteristics of the Isolates

Biological characterization of the potential probiotic isolates was conducted, including growth and acid production capacity, tolerance to low pH and bile salt, and antagonistic pathogenic bacteria, which were the main features of potential probiotics. All tests were performed three times. The potential probiotics were grown separately in 10 mL of MRS broth media for 24 h at 37 °C for further experimentation.

#### 2.3.1. The Growth and Acid Production Capacity

MRS broth was inoculated with 1% probiotics and cultured at 37 °C and 180 rpm for 4 h, 8 h, 12 h, 16 h, 20 h, and 24 h, respectively. The growth capacity of isolates was determined by measuring turbidity of culture media using a spectrophotometer (752N, INESA, Shanghai, China) at 600 nm. Similarly, the acid production capacity of isolates was detected by measuring the pH of the medium using a pH meter (ST20, OHAUS, Parsippany, NJ, USA).

#### 2.3.2. Tolerance to Low pH

MRS broths at pH 2.0, pH 3.0, pH 4.0, pH 5.0, and pH 6.0 were inoculated with 1% isolated strains and incubated at 37 °C for 24 h, respectively. The survival of probiotics at different pH levels was measured using a spectrophotometer at 600 nm, and the pH 6.0 medium was used as the control to calculate the survival rate, as follows:

Survival rate (%) = N_x_/N_0_ × 100% (1)

N_x_ is the absorbance after 24 h of culture in different pH media. N_0_ is the absorbance after 24 h of culture in a pH 6.0 medium.

#### 2.3.3. Tolerance to Bile Salt

MRS broths supplemented with 0.1%, 0.15%, 0.2%, 0.25%, and 0.3% (*w*/*v*) of bile salt (pig) were inoculated with 1% isolates and incubated at 37 °C for 24 h, respectively. The survival of probiotics at different bile salt levels was measured using a spectrophotometer at 600 nm, and the medium with no bile salt was used as the control to calculate the survival rate, as follows:

Survival rate (%) = N_x_/N_0_ × 100% (2)

N_x_ is the absorbance after 24 h of culture in different bile salt concentration media. N_0_ is the absorbance after 24 h of culture in bile salt-free medium.

#### 2.3.4. Antimicrobial Activity

Pathogenic strains, including *Staphylococcus aureus*, *Salmonella gallinarum*, and *Escherichia coli* (*E. coli*), were used as test organisms. A volume of 100 μL overnight culture of each indicator bacterium was swabbed evenly over the surface of a nutrient agar plate with sterile triangular glass rods. The Oxford cups with upright position were placed on the dried plate, and 200 μL of the isolated bacteria was added to each well and incubated at 37 °C for 24 h. The diameter of the inhibition zone around the well was measured to determine the antibacterial ability of the probiotics.

### 2.4. Effects of Isolated Probiotics on the Production and Immunity of Pekin Ducks

A total of 90 healthy 7-day-old Pekin ducks were separated into 3 groups randomly (6 replicates in each group and 5 ducks in each replicate). The ducks in the LP group and EF group were fed 1 × 10^7^ CFU/mL of *L. plantarum* and *E. faecalis* in drinking water, respectively. The control group ducks were allowed to drink water without probiotics. All ducks had free access to feed and water (Table 1). The routine husbandry management was carried out through the experiments.

#### 2.4.1. Analysis of the Growth and Slaughter Performance

The body weight and feed consumption of each duck were recorded every week, and the average daily feed intake (ADFI), average daily gain (ADG), and feed conversion ratio (FCR) were calculated. Six ducks were randomly selected from each group and weighed individually at 42 d (before slaughter) after 24 h of fasting. After slaughter, the carcass weight, half-eviscerated weight, eviscerated weight, breast muscle weight, and leg muscle weight were measured, and the indicators, including percentage of half-eviscerated yield, percentage of eviscerated yield, percentage of breast, and percentage of leg muscle, were calculated.

#### 2.4.2. Histomorphological Examination and RT-PCR for Small Intestine

The small intestinal tissues of 3 groups, including duodenum, jejunum, and ileum, were collected. One part of the small intestine was used for histomorphological determination, and the other part was used for RT-PCR of immune-related genes.

The duodenum, jejunum, and ileum were washed in ice-cold saline and fixed in 4% polyformaldehyde solution for histomorphology determination. Then, the samples were washed under running water, dehydrated with an ethyl alcohol series, cleared in xylene, and embedded in paraffin wax. The slices of 6 μm thickness were cut and stained with hematoxylin–eosin staining (H&E staining). The villus height and crypt depth of the small intestine were measured using a microscope photography system (Olympus, Tokyo, Japan), and the villus height and crypt depth ratio (V/C) was calculated. Five fields of each slice were randomly selected for statistical analysis, and an average was taken.

The RNA of duodenum, jejunum, and ileum was extracted with Trizol reagent (Invitrogen, Carlsbad, CA, USA) according to the manufacturer’s instructions. The RNA concentration was detected by Nanodrop 2000 (Thermo Fisher Scientific, Waltham, CA, USA), and quality was measured using 1% agarose electrophoresis. Then, the first strand of cDNA was synthesized according to PrimeScript^TM^ RT Reagent Kit with gDNA Eraser (TaKaRa, Dalian, China), and the amplification was performed with an SYBR Premix Ex Taq II (Takara, Kyoto, Japan) by a CFX Opus 96 Real-Time PCR System (Bio-Rad, Hercules, CA, USA). The primers for duck immune-related genes interferon-gamma (*INF-γ*), mucin2 (*MUC2*), interleukin 6 (*IL-6*), and the housekeeping gene *β-actin* are shown in Table 2. All reactions contained 3 technical replicates, and gene expression level was analyzed using the 2^−∆∆CT^ method [20].

#### 2.4.3. Content of Serum IgG and IgA

The blood was collected from the duck wing vein (from the ducks sampled above). After standing at room temperature for 2 h, the serum samples were obtained by centrifuging at 4000 rpm for 10 min and stored at −20 °C. The content of serum immunoglobulin A (IgA) and immunoglobulin G (IgG) was measured according to the instructions of the ELISA kits (Jiancheng, Nanjing, China).

#### 2.4.4. Changes in Cecal Microflora

The total bacterial DNA of three duck groups was extracted according to the manufacturer’s instructions (Solarbio, Beijing, China). The ratios of 260 nm/280 nm and 260 nm/230 nm were used as an indicator of both DNA quality and quantity. According to the quantitative primers of the 16S rRNA gene sequence of *Escherichia coli*, *Helicobacter pullorum*, *Campylobacter* sp., *Salmonella* sp., and *Lactobacillus* sp. (Table 2), each bacterial fragment was amplified using PCR. The fragment was mixed with a pMD™19-T cloning vector (TaKaRa, Dalian, China), ligated overnight at 16 °C, and then the plasmid was transferred into DH5α competent *E. coli*.

DH5α was swabbed evenly over the surface of a Luria–Bertani (LB) broth with ampicillin and cultured at 37 °C for 24 h. The colony PCR was performed, and the product was examined by 1.5% agarose gel electrophoresis, purified, and sequenced. Then, the plasmid of each bacterium was extracted according to the instructions (OMEGA, Norcross, GA, USA). According to the molecular weight and mass concentration of the plasmid, the copy number was calculated to make the standard product. The calculation formula is as follows: copies/μL = (6.02 × 10^23^ copy number/mol) × (plasmid concentration ng/μL × 10^−9^)/(plasmid base number × 660/mol). The standard product was diluted according to a 10 times the gradient ratio (diluting 8 gradients), and 2 μL of each gradient was used as a template for RT-PCR amplification. Based on the results, a standard curve was established with the logarithm of plasmid copy number as the *x*-axis and Ct value as the *y*-axis. Then, the RT-PCR was performed, and the copy number of each strain in the sample was calculated according to the standard curve and the Ct value of the sample. Each sample was analyzed in triplicate.

### 2.5. Statistical Analysis

All data were presented as mean ± SD, percentage, and figures. Statistical analyses of the data were conducted in SPSS 18.0 software (version 1.0). Significant differences among treatments were determined by one-way ANOVA followed by Tukey’s test and Duncan’s test with a level of significance at *p* < 0.05.

## 3. Results

### 3.1. Isolation and Identification of Bacterial Strains

**Figure 1:**
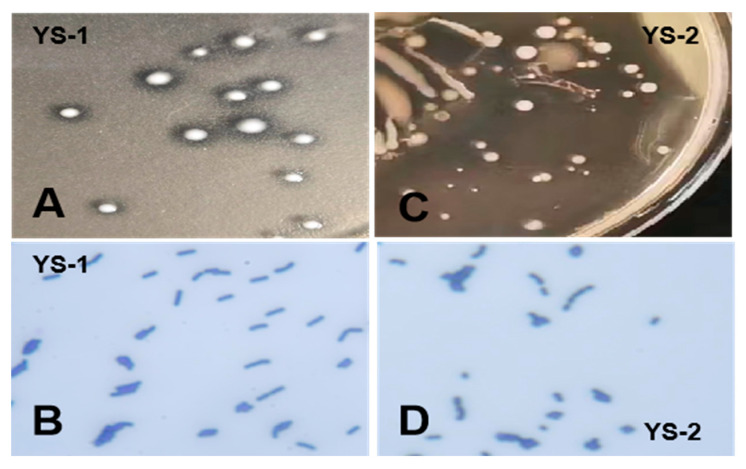
Morphology of bacterial strains. (**A**) Colony morphology of YS-1. (**B**) Gram staining of YS-1. (**C**) Colony morphology of YS-2. (**D**) Gram staining of YS-2.

In this study, two bacterial species (named YS-1 and YS-2) with probiotic potential were isolated from the cecum of Pekin ducks. The colony morphology showed that YS-1 was white, round, with a neat edge and a calcium-soluble circle; YS-2 was white, round, with a moist and opaque surface, and a neat edge. Both YS-1 and YS-2 showed positive results by Gram staining, among which YS-1 was rod-shaped bacteria and YS-2 was coccus-shaped bacteria (Figure 1). The results of bacterial micro-biochemical identification, including a catalase test, V-P test, MR-VP test, gelatin liquefaction test, hydrogen sulfide test, and complete biochemical identification test of lactic acid bacteria, also indicated that YS-1 and YS-2 were positive in biochemical tests. The 16s rDNA sequencing showed that YS-1 and YS-2 were 100% similar to *L. plantarum* and *E. faecalis*, respectively (Table S1).

### 3.2. Biological Characteristics of the Probiotics

**Figure 2:**
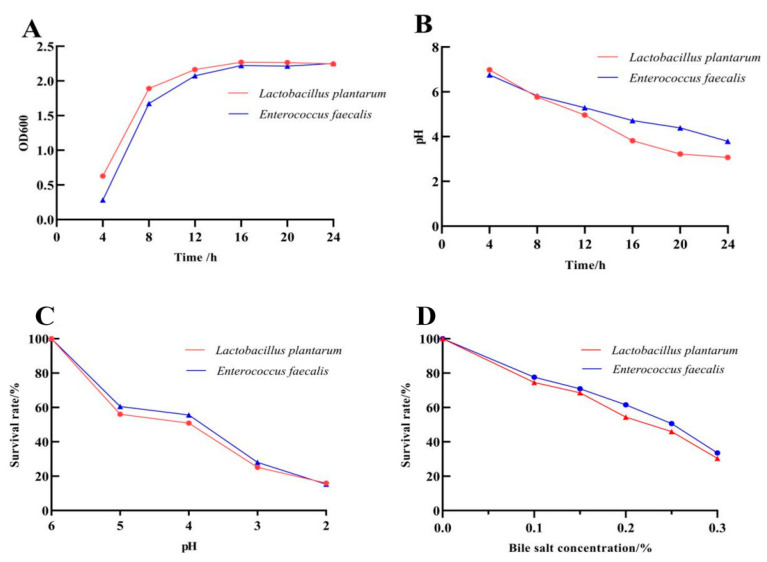
Biological characteristics of the probiotics. (**A**) Growth capacity curve. (**B**) Acid production capacity curve. (**C**) Acid tolerance curve. (**D**) Bile salt tolerance curve.

*L. plantarum* and *E. faecalis* were in the logarithmic growth stage from 4 to 16 h, with rapid reproduction, indicating good growth characteristics. Moreover, with the passage of time, the two strains produced acid during their growth process, and the pH decreased from about 7.0 to around 3.5, suggesting that they had good acid-producing capacity. After 24 h of exposure at pH 3, *L. plantarum* and *E. faecalis* showed high survival rates of (25.10 ± 0.49)% and (28.04 ± 0.46)%, respectively, suggesting the isolates exhibited high tolerance to low pH. The survival rate of *L. plantarum* and *E. faecalis* was reduced when the bile salt concentration was increased. Both strains showed a high level of tolerance to a bile salt concentration of 0.3 % after exposure for 24 h, with survival rates of (30.36 ± 0.38)% and (33.51 ± 0.29)%, respectively (Figure 2).

Both *L. plantarum* and *E. faecalis* were able to inhibit the growth of pathogenic bacteria such as *Staphylococcus aureus*, *Salmonella gallinarum*, and *Escherichia coli* in this study. The inhibition zones of *L. plantarum* and *E. faecalis* against pathogenic bacteria were all greater than 12 mm (Table 3). Therefore, *L. plantarum* and *E. faecalis* had good biological characteristics.

### 3.3. Effects of L. plantarum and E. faecalis on Production and Immunity of Pekin Duck

Compared to the control group, the addition of *L. plantarum* and *E. faecalis* significantly increased the ADFI and ADG of Pekin ducks (*p* < 0.05) and improved the live weight, eviscerated weight, half-eviscerated weight, breast muscle weight, and leg muscle weight (*p* < 0.05). The FCR was not affected by the supplementation of *L. plantarum* and *E. faecalis* in drinking water, and no significant differences were found in the carcass weight between the test groups and the control group either (*p* > 0.05) (Table 4).

A significant increase was shown in the duodenal villus height, duodenal V/C, and ileal villus height of ducks drinking water with *L. plantarum* and *E. faecalis* in comparison with the control group (*p* < 0.05). No significant differences were found in duodenal crypt depth, ileal crypt depth, or ileal V/C, nor in the villus height, crypt depth, or V/C of jejunum between the experimental groups and control group (*p* > 0.05) (Table 5).

**Figure 3:**
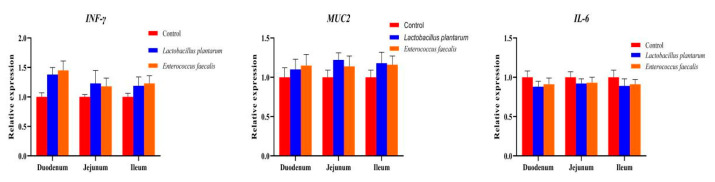
Relative expression levels of *INF-γ*, *MUC2,* and *IL-6* in the duodenum, jejunum, and ileum of Pekin ducks.

Compared with the ducks in the control group, the bursa index and spleen index were significantly increased in the ducks drinking the *L. plantarum* and *E. faecalis* (*p* < 0.05). Despite the slight increase in the thymus index, there were no significant differences between the probiotics-treated groups and the control group (*p* > 0.05). The serum IgG levels in the LP group and EF group were significantly decreased, while the serum IgA levels in the two experimental groups were significantly increased compared with the control (*p* < 0.05) (Table 5). Moreover, no significant differences were observed in the relative expression levels of *INF-γ*, *IL-6,* and *MUC2* in the duodenum, jejunum, and ileum between the ducks of the two experiment groups and the control group (*p* > 0.05) (Figure 3). These results indicated that *L. plantarum* and *E. faecalis* can improve the production and immune performance of ducks.

### 3.4. Effects of L. plantarum and E. faecalis on the Intestinal Flora of Pekin Ducks

The similarity between the PCR product sequences of 5 strains (*Escherichia coli*, *Helicobacter pullorum*, *Campylobacter* sp., *Salmonella* sp., and *Lactobacillus* sp.) and the strain sequences provided in NCBI was higher than 95%. The correlation coefficient of the standard curves for each strain was above 0.9883, and the amplification efficiency ranged from 98.8% to 107.5% (Table 6).

RT-PCR for *Escherichia coli*, *Helicobacter pullorum*, *Campylobacter* sp., *Salmonella* sp., and *Lactobacillus* sp. was performed, and the results showed that adding *L. plantarum* and *E. faecalis* significantly decreased the numbers of *Escherichia coli*, *Campylobacter* sp., and *Salmonella* sp. of ducks in comparison with the control group (*p* < 0.05). However, despite a slight decrease, there were no significant differences in the number of *Helicobacter pullorum* between the experiment and control groups (*p* > 0.05). Moreover, the quantity of *Lactobacillus* sp. in LP and EF groups was extremely significantly higher than that in the control group (*p* < 0.01), indicating that *Lactobacillus* sp. were the dominant bacteria in the two experimental groups (Table 7).

## 4. Discussion

Probiotics could directly adhere to or degrade harmful substances through themselves and their metabolites or exert indirect probiotic effects by regulating the intestinal flora or metabolic enzyme activity of flora, host immune activity, and part of enzyme activity [30,31,32]. Therefore, supplementing probiotics to regulate the gut microbiota and improve host growth and immune function has become a popular method in the international livestock industry, especially in improving individual gastrointestinal digestion and absorption as well as related diseases. Studies have shown that *Lactobacillus salivarius* can act as a potential probiotic to improve growth performance, fecal microbiota, and the immune response of chickens [33]. The addition of *L. plantarum* can also enhance the growth performance and intestinal health of Pekin ducks [34]. In recent years, there have been many studies on probiotics, and the efficacy of probiotics has shown obvious strain specificity and individual differences. Therefore, screening, identifying, and further promoting the development and utilization of probiotics in production were of great significance for improving the production and immune performance of ducks and promoting the efficient development of duck farming.

*L. plantarum* and *E. faecalis* were both probiotics and played important roles in maintaining intestinal health. *L. plantarum* can be used as a fermentation agent for food and was also a gastrointestinal probiotic that can adhere to and colonize on the intestinal mucosa, playing a key role in the competitive elimination of pathogenic bacteria [35,36]. *E. faecalis*, a facultative anaerobic bacterium belonging to the *Enterococcus* family in the order *Lactobacillales*, had strong tolerance to animal gastric fluid, intestinal fluid, and bile salts. It enhanced animal growth performance, maintained gastrointestinal microbiota balance, and strengthened the body’s resistance to diseases by producing extracellular polysaccharides to increase intestinal adhesion and promote probiotic colonization [37,38]. In this study, two isolates were screened from the cecum of Pekin ducks. Through colony morphology observation, biochemical identification, and 16S rDNA sequence analysis, the two strains were identified as *L. plantarum* and *E. faecalis*, respectively.

The growth and acid-producing capacity of lactic acid bacteria are important indicators for evaluating the metabolic ability of bacterial strains, and the gastrointestinal digestion tolerance of lactic acid bacteria is also used to evaluate whether they have potential probiotic properties. The growth of lactic acid bacteria will be inhibited after being affected by gastric acid and bile salt, so the survival of the strain in the gastrointestinal tract is the basis of determining its probiotic properties. The pH of the gastric juices of birds has been reported to be an average of 3.5 [39]. In this experiment, *L. plantarum* and *E. faecalis* showed good growth ability, acid production, acid resistance, and bile salt resistance, indicating that they had strong probiotic properties and exert probiotic functions in the gastrointestinal tract. Meanwhile, the two strains were able to inhibit the growth of *Escherichia coli*, *Staphylococcus aureus,* and *Salmonella gallinarum*, suggesting that they may be used as feed additives to improve the antibacterial ability of animals and reduce the occurrence of diseases.

Probiotic strains discovered from natural hosts were more desirable than isolates obtained from other sources due to their beneficial effects on host organisms and safety records, and it was the most promising probiotic for poultry nutrition [40]. In animal production, lactic acid bacteria were mainly used as probiotic feed additives, which had the functions of improving intestinal microbiota balance, promoting growth, and enhancing immunity. Studies have shown that *L. plantarum* GX17 was beneficial for the growth performance and improvement in intestinal barrier/gut microbiota function in yellow-feathered broiler chickens [41]. *E. faecalis*-1, isolated from healthy chickens, could promote the growth and immune performance of broiler chickens and regulate the cecal microbiota [42]. In this study, the ADFI, ADG, live weight, eviscerated weight, half-eviscerated weight, breast muscle weight, and leg muscle weight in the LP group and EF group were significantly higher than the control group, indicating that the isolated *L. plantarum* and *E. faecalis* could improve the growth and slaughter performance of Pekin ducks. Intestinal villus height and crypt depth were the important indicators that affected the digestion and absorption of nutrients, which determined the intestinal absorption capacity of animals. The addition of *L. plantarum* and *E. faecalis* in this experiment significantly increased the villus height and V/C of the duodenum in Pekin ducks, as well as the villus height of the ileum, suggesting that *L. plantarum* and *E. faecalis* could promote the digestion and absorption of nutrients in the duck intestine. Therefore, the addition of *L. plantarum* and *E. faecalis* could improve the production performance of Pekin ducks.

IgA and IgG played a key role in protecting the body from attack by infectious microorganisms [43,44]. Moreover, the genes *INF-γ*, *MUC2,* and *IL-6* have been shown to play important roles in the body’s immune functions [45,46]. The levels of serum IgA and IgG, as well as the relative expression levels of the intestinal immune-related genes *INF-γ*, *MUC2,* and *IL-6*, were important indicators for detecting the immunomodulatory effects of *Lactobacillus* on the body. The results of this study showed that the supplementation of *L. plantarum* and *E. faecalis* in drinking water could significantly increase the immune organ index and serum IgA content and decrease the serum IgG content of Pekin ducks. At the same time, the relative expression levels of *INF-γ* and *MUC2* in the duodenum, jejunum, and ileum were increased, and the relative expression levels of *IL-6* were decreased. These results indicated that *L. plantarum* and *E. faecalis* could enhance the immune performance of Pekin ducks.

The process of intestinal digestion was closely related to the gut microbiota, such as nutrient absorption, feed digestibility, energy harvest, and productivity, all of which were influenced by the composition and diversity of the microbiota [47,48]. Intestinal pathogens and their toxins can disrupt the intestinal barrier in animals. Disruption of the dynamic balance of animal gut microbiota can lead to impaired digestion and immune function, as well as increased susceptibility to pathogens, resulting in diarrhea and decreased growth performance [49,50]. Some studies showed that the duck-derived lactic acid bacteria can improve the growth performance and meat quality of Muscovy ducks by regulating intestinal morphology and microbial community [51]. Similarly, supplementation of *Lactobacillus acidophilus* and *Bacillus subtilis* could significantly change the growth performance, serum immunity, and cecal microbiota of Cherry Valley ducks during the fattening period [52]. In this study, the addition of *L. plantarum* and *E. faecalis* could reduce the number of pathogenic bacteria, such as *Escherichia coli*, *Campylobacter* sp., *Salmonella* sp., and *Helicobacter pullorum,* in the cecum of Pekin ducks and increase the content of *Lactobacillus* sp., so as to improve the structure of intestinal microflora and promote intestinal microecological balance.

## 5. Conclusions

In summary, two strains were isolated from the cecum of Pekin ducks in this experiment and identified as *L. plantarum* and *E. faecalis* by colony morphological observation, biochemical identification, and 16S rDNA sequence analysis. Both strains had good biological characteristics. The supplementation of *L. plantarum* and *E. faecalis* in drinking water can improve the production and slaughter performance of Pekin ducks, as well as enhance their immune levels. In addition, they can also reduce the number of pathogenic bacteria in the cecum and maintain the stability of intestinal flora. These findings indicated that duck-derived *L. plantarum* and *E. faecalis* may be used as probiotic feed additives to promote the intestinal health of Pekin ducks and improve their production and immune performance. Due to the small sample size, there is not yet sufficient evidence to prove the effectiveness of duck-derived probiotics. In the future, we will increase the number of experimental ducks to more accurately determine the application effect of duck-derived probiotics.

## Figures and Tables

**Table 1:** The feed composition of Pekin duck.

Ingredient	Content (%)	Nutrient	Content (%)
Corn	56.00	Avian metabolizable energy	2875 Mcal·kg^−1^
Soybean meal	23.80	Crude protein	15.700
Corn gluten meal	10.00	Calcium	0.900
Limestone	7.00	Total phosphorus	0.680
CaHPO_4_	1.50	Available phosphorus	0.450
Premix	1.00	Salt	0.370
NaCl	0.30	Lysine	0.760
Lys·HCl	0.30	Methionine	0.387
DL-Met	0.10	Methionine + Cystine	0.654
Total	100.00	Isoleucine	0.534
		Threonine	0.579
		Tryptophan	0.194
		Crude fiber	4.100
		Crude fat	3.400
		Crude ash	5.200

**Table 2:** Primer sequences for *INF-γ*, *MUC2*, *IL-6* and bacterial strains.

Name (Target Gene)	Primer (5′-3′)	Length	Reference
*INF-γ*	F:GCTGATGGCAATCCTGTTTT	247 bp	[21]
	R:GGATTTTCAAGCCAGTCAGC		
*MUC2*	F:AGTTCTTGCCTAATTCCTCAGTCT	146 bp	[22]
	R:TTGCCGTTCATATCCAGGTTCA		
*IL-6*	F:TTCGACGAGGAGAAATGCTT	150 bp	[23]
	R:CCTTATCGTCGTTGCCAGAT		
*β-actin*	F:CCCTGTATGCCTCTGGTCG	194 bp	[24]
	R:CTCGGCTGTGGTGGTGAAG		
*Escherichia coli* (*irp2*)	F: GCTCTGTGCCCTTTGA	261 bp	[25]
	R: GGCGGGAGGAGTAGTT		
*Helicobacter pullorum* (*gyrA*)	F: CAAGAATCGTGGGTGATG	351 bp	[26]
	R: GTGGAATATTTGTCGCCA		
*Campylobacter* sp. (*aicsxii*)	F: CACATTAAATCTTTATTTTCAACCCGCTGAA	73 bp	[27]
	R: ACAATCCATCTTCTATCATTGCCTTAGC		
*Salmonella* sp. (*invA*)	F: CAATGGCGGCGAATTACGAG	100 bp	[28]
	R: AAGGCTGAGGAAGGTACTGC		
*Lactobacillus* sp.	F: CGATGAGTGCTAGGTGTTGGA	186 bp	[29]
	R: CAAGATGTCAAGACCTGGTAAG		

**Table 3:** Antimicrobial activity of *L. plantarum* and *E. faecalis* from duck cecum against pathogens.

Name	Strain and Diameter of Antibacterial Zone (mm)		
	*Staphylococcus aureus*	*Escherichia coli*	*Salmonella gallinarum*
*L. plantarum*	12.20 ± 0.92	12.92 ± 0.74	13.42 ± 1.40
*E. faecalis*	13.92 ± 2.12	12.48 ± 0.23	14.07 ± 2.41

**Table 4:** Effects of *L. plantarum* and *E. faecalis* on growth and slaughter performance of Pekin ducks.

Performance	Control Group	LP Group	EF Group
ADFI (g/d)	251.77 ± 3.89 ^b^	259.34 ± 4.32 ^a^	257.18 ± 4.18 ^b^
ADG (g/d)	110.32 ± 2.04 ^b^	118.15 ± 1.84 ^a^	116.58 ± 1.99 ^a^
FCR	2.28 ± 0.22	2.20 ± 0.23	2.21 ± 0.26
Live weight (g)	3278.74 ± 30.76 ^b^	3462.15 ± 47.85 ^a^	3432.69 ± 40.32 ^a^
Carcass weight (g)	2866.32 ± 27.55	2902.38 ± 35.69	2887.65 ± 34.82
Eviscerated weight (g)	2401.58 ± 35.83 ^b^	2573.46 ± 33.28 ^a^	2568.14 ± 34.49 ^a^
Half-eviscerated weight (g)	2698.14 ± 40.48 ^b^	2832.08 ± 38.77 ^a^	2828.78 ± 39.21 ^a^
Breast muscle weight (g)	231.23 ± 8.54 ^b^	245.55 ± 9.85 ^a^	242.14 ± 9.42 ^a^
Leg muscle weight (g)	266.12 ± 7.15 ^b^	282.27 ± 8.56 ^a^	289.32 ± 8.58 ^a^

Note: Same superscript within a row indicates no significant differences (*p* > 0.05), different superscripts of lowercase letters within a row indicate significant differences (*p* < 0.01), and different superscripts of capital letters within a row indicate significant differences (*p* < 0.05). The same below.

**Table 5:** Effects of *L. plantarum* and *E. faecalis* on Immune Organ Index and Serum IgG, IgA Content in Pekin Ducks.

Items	Control Group	LP Group	EF Group
Villus height in duodenum (μm)	1007.35 ± 71.33 ^b^	1107.54 ± 50.05 ^a^	1101.21 ± 53.26 ^a^
Crypt depth in duodenum (μm)	207.78 ± 20.26	213.98 ± 25.45	208.85 ± 31.23
V/C in duodenum	4.85 ± 0.33 ^b^	5.18 ± 0.42 ^a^	5.27 ± 0.40 ^a^
Villus height in jejunum (μm)	637.65 ± 45.01 ^b^	656.56 ± 37.75 ^a^	650.50 ± 38.44 ^a^
Crypt depth in jejunum (μm)	208.42 ± 18.83	211.23 ± 21.36	210.38 ± 24.45
V/C in jejunum	3.06 ± 0.35	3.11 ± 0.22	3.09 ± 0.33
Villus height in ileum (μm)	664.83 ± 87.35 ^b^	679.88 ± 56.58 ^a^	677.67 ± 67.43 ^a^
Crypt depth in ileum (μm)	155.90 ± 20.63	158.54 ± 24.08	156.38 ± 23.15
V/C in ileum	4.26 ± 0.25	4.29 ± 0.38	4.33 ± 0.29
Thymus index (%)	1.71 ± 0.21	1.78 ± 0.16	1.75 ± 0.16
Bursa index (%)	0.62 ± 0.11 ^b^	0.70 ± 0.09 ^a^	0.72 ± 0.10 ^a^
Spleen index (%)	0.57 ± 0.07 ^b^	0.64 ± 0.05 ^a^	0.63 ± 0.08 ^a^
Serum IgG content (μg/mL)	752.79 ± 42.34 ^a^	679.08 ± 48.25 ^b^	684.23 ± 36.12 ^b^
Serum IgA content (μg/mL)	123.28 ± 12.48 ^b^	136.78 ± 15.87 ^a^	137.74 ± 13.75 ^a^

**Table 6:** Standard curves for RT-PCR of various bacterial strains.

Strains	Standard Curve	Correlation Coefficient (R^2^)	Amplification Efficiency (E)
*Escherichia coli*	y = −2.8283x + 24.113	0.9883	102.3 %
*Helicobacter* *pullorum*	y = −2.9817x + 28.337	0.9956	107.5 %
*Campylobacter* sp.	y = −3.0549x + 28.961	0.9913	100.1 %
*Salmonella* sp.	y = −3.1228x + 33.136	0.9942	98.8 %
*Lactobacillus* sp.	y = −3.2248x + 31.456	0.9938	100.9 %

**Table 7:** Effects of *L. plantarum* and *E. faecalis* on cecal microflora of Pekin ducks.

Name	Control Group	LP Group	EF Group
*Escherichia coli*	5.28 ± 0.13 ^a^	4.72 ± 0.08 ^b^	4.49 ± 0.11 ^b^
*Helicobacter pullorum*	3.73 ± 0.11	3.64 ± 0.08	3.58 ± 0.09
*Campylobacter* sp.	4.27 ± 0.27 ^a^	3.84 ± 0.15 ^b^	4.02 ± 0.23 ^b^
*Salmonella* sp.	4.79 ± 0.20 ^a^	3.53 ± 0.35 ^b^	3.77 ± 0.45 ^b^
*Lactobacillus* sp.	3.66 ± 0.22 ^B^	5.38 ± 0.29 ^A^	4.86 ± 0.36 ^A^

## Data Availability

Data are contained within the article and Supplementary Materials.

## Supplementary Materials

The following supporting information can be downloaded at: https://www.mdpi.com/article/10.3390/biom15091217/s1, Table S1: The sequences of YS-1 and YS-2.
