# Supplementary material for: Isolation and Identification of Duck Intestinal Probiotics and Their Effects on the Production and Immune Performance of Pekin Ducks
Source: Biomolecules. 2025 Aug 22;15(9):1217. doi: 10.3390/biom15091217 (PMC12467036; doi:10.3390/biom15091217)
Supplement: Supplementary file 1 [file biomolecules-15-01217-s001.zip › biomolecules-3787743-supplementary.pdf]

Table S1 The sequences of YS-1 and YS-2

	Squences (5’-3’)
YS-1	GCAGTCGAACGAACTCTGGTATTGATTGGTGCTTGCATCATGATTTACATTTGAGTGAGTGGCGAACTGGTGAGTAACACGTGGGAAACCTG<br>CCCAGAAGCGGGGGATAACACCTGGAAACAGATGCTAATACCGCATAACAACTTGGACCGCATGGTCCGAGCTTGAAAGATGGCTTCGGCT<br>ATCACTTTTGGATGGTCCCGCGGCGTATTAGCTAGATGGTGGGGTAACGGCTCACCATGGCAATGATACGTAGCCGACCTGAGAGGGCAATC<br>GGCCACATTGGGACTGAGACACGGCCCAAACTCCTACGGGAGGCAGCAGTAGGGAATCTTCCACAATGGACGAAAGTCTGATGGAGCAAC<br>GCCGCGTGAGTGAAGAAGGGTTTCGGCTCGTAAAACTCTGTTGTTAAAGAAGAACATATCTGAGAGTAACTGTTCAGGTATTGACGGTATTT<br>AACCAGAAAGCCACGGCTAACTACGTGCCAGCAGCCGCGGTAATACGTAGGTGGCAAGCGTTGTCCGGATTTATTGGGCGTAAAGCGAGCG<br>CAGGCGGTTTTTTAAGTCTGATGTGAAAGCCTTCGGCTCAACCGAAGAAGTGCATCGGAAACTGGGAAACTTGAGTGCAGAAGAGGACAG<br>TGGAACTCCATGTGTAGCGGTGAAATGCGTAGATATATGGAAGAACACCAGTGGCGAAGGCGGCTGTCTGGTCTGTAACTGACGCTGAGGC<br>TCGAAAGTATGGGTAGCAAACAGGATTAGATACCCTGGTAGTCCATACCGTAAACGATGAATGCTAAGTGTTGGAGGGTTTCCGCCCTTCAG<br>TGCTGCAGCTAACGCATTAAGCATTCCGCCTGGGGAGTACGGCCGCAAGGCTGAAACTCAAAGGAATTGACGGGGGCCCGCACAAGCGGT<br>GGAGCATGTGGTTTAATTCGAAGCTACGCGAAGAACCTTACCAGGTCTTGACATACTATGCAAATCTAAGAGATTAGACGTTCCCTTCGGGG<br>ACATGGATACAGGTGGTGCATGGTTGTCGTCAGCTCGTGTCGTGAGATGTTGGGTTAAGTCCCGCAACGAGCGCAACCCTTATTATCAGTTG<br>CCAGCATTAAGTTGGGCACTCTGGTGAGACTGCCGGTGACAAACCGGAGGAAGGTGGGGATGACGTCAAATCATCATGCCCCTTATGACCT<br>GGGCTACACACGTGCTACAATGGATGGTACAACGAGTTGCGAACTCGCGAGAGTAAGCTAATCTCTTAAAGCCATTCTCAGTTCGGATTGTA<br>GGCTGCAACTCGCCTACATGAAGTCGGAATCGCTAGTAATCGCGGATCAGCATGCCGCGGTGAATACGTTCCCGGGCCTTGTACACACCGCC<br>CGTCACACCATGAGAGTTTGTAACACCCAAAGTCGGTGGGGTAACCTTTTAGGAACCAGCCGCC
YS-1	TGCAGTCGAACGCTTCTTTCCTCCCGAGTGCTTGCACTCAATTGGAAAGAGGAGTGGCGGACGGGTGAGTAACACGTGGGTAACCTACCCA<br>TCAGAGGGGGATAACACTTGGAAACAGGTGCTAATACCGCATAACAGTTTATGCCGCATGGCATAAGAGTGAAAGGCGCTTTCGGGTGTCG<br>CTGATGGATGGACCCGCGGTGCATTAGCTAGTTGGTGAGGTAACGGCTCACCAAGGCCACGATGCATAGCCGACCTGAGAGGGTGATCGGC<br>CACACTGGGACTGAGACACGGCCCAGACTCCTACGGGAGGCAGCAGTAGGGAATCTTCGGCAATGGACGAAAGTCTGACCGAGCAACGC<br>CGCGTGAGTGAAGAAGGTTTTCGGATCGTAAAACTCTGTTGTTAGAGAAGAACAAGGACGTTAGTAACTGAACGTCCCCTGACGGTATCTA<br>ACCAGAAAGCCACGGCTAACTACGTGCCAGCAGCCGCGGTAATACGTAGGTGGCAAGCGTTGTCCGGATTTATTGGGCGTAAAGCGAGCGC

AGGCGGTTTCTTAAGTCTGATGTGAAAGCCCCCGGCTCAACCGGGGAGGGTCATTGGAAACTGGGAGACTTGAGTGCAGAAGAGGAGAGT
GGAATTCCATGTGTAGCGGTGAAATGCGTAGATATATGGAGGAACACCAGTGGCGAAGGCGGCTCTCTGGTCTGTAACTGACGCTGAGGCT
CGAAAGCGTGGGGAGCAAACAGGATTAGATACCCTGGTAGTCCACGCCGTAAACGATGAGTGCTAAGTGTTGGAGGGTTTCCGCCCTTCAG
TGCTGCAGCAAACGCATTAAGCACTCCGCCTGGGGAGTACGACCGCAAGGTTGAAACTCAAAGGAATTGACGGGGGCCCGCACAAGCGGT
GGAGCATGTGGTTTAATTCGAAGCAACGCGAAGAACCTTACCAGGTCTTGACATCCTTTGACCACTCTAGAGATAGAGCTTTCCCTTCGGGG
ACAAAGTGACAGGTGGTGCATGGTTGTCGTCAGCTCGTGTCGTGAGATGTTGGGTTAAGTCCCGCAACGAGCGCAACCCTTATTGTTAGTT
GCCATCATTTAGTTGGGCACTCTAGCGAGACTGCCGGTGACAAACCGGAGGAAGGTGGGGATGACGTCAAATCATCATGCCCCTTATGACC
TGGGCTACACACGTGCTACAATGGGAAGTACAACGAGTCGCTAGACCGCGAGGTCATGCAAATCTCTTAAAGCTTCTCTCAGTTCGGATTGC
AGGCTGCAACTCGCCTGCATGAAGCCGGAATCGCTAGTAATCGCGGATCAGCACGCCGCGGTGAATACGTTCCCGGGCCTTGTACACACCG
CCCGTCACACCACGAGAGTTTGTAACACCCGAAGTCGGTGAGGTAACCTTTTGGAGCCAGCCGCCTAA
